# Hydrothermal Synthesis of Various Hierarchical ZnO Nanostructures and Their Methane Sensing Properties

**DOI:** 10.3390/s130506170031

**Published:** 2013-05-10

**Authors:** Qu Zhou, Weigen Chen, Lingna Xu, Shudi Peng

**Affiliations:** 1 State Key Laboratory of Power Transmission Equipment & System Security and New Technology, Chongqing University, Chongqing 400030, China; E-Mail: lingnaxu@cqu.edu.cn; 2 Chongqing Electric Power Research Institute, Chongqing 401123, China; E-Mail: psdzq@yahoo.cn

**Keywords:** hierarchical nanostructures, ZnO gas sensor, growth mechanism, methane, sensing properties

## Abstract

Hierarchical flower-like ZnO nanorods, net-like ZnO nanofibers and ZnO nanobulks have been successfully synthesized via a surfactant assisted hydrothemal method. The synthesized products were characterized by X-ray powder diffraction and field emission scanning electron microscopy, respectively. A possible growth mechanism of the various hierarchical ZnO nanostructures is discussed in detail. Gas sensors based on the as-prepared ZnO nanostructures were fabricated by screen-printing on a flat ceramic substrate. Furthermore, their gas sensing characteristics towards methane were systematically investigated. Methane is an important characteristic hydrocarbon contaminant found dissolved in power transformer oil as a result of faults. We find that the hierarchical flower-like ZnO nanorods and net-like ZnO nanofibers samples show higher gas response and lower operating temperature with rapid response-recovery time compared to those of sensors based on ZnO nanobulks. These results present a feasible way of exploring high performance sensing materials for on-site detection of characteristic fault gases dissolved in transformer oil.

## 1. Introduction

Power transformers are the most important and costly components in power transmission or distribution substations [1,2]. Their operating states are closely related to the security, reliability and economy of the whole power system [3]. Presently, most power transformers still employ oil-paper insulation. Once faults have happened in a power transformer due to aging, overheating or discharge, some low molecular weight characteristic fault gases are generated and dissolve in the transformer oil [4,5]. It has been recognized for many years that online monitoring of the concentrations and rates of generation of these gases is one of the most effective methods for transformer condition assessment and fault diagnosis [6], so rapid, facile and accurate detection of these fault characteristic gases is especially important and currently the subject of intensive research. Gas sensing technology is the critical part in an on-line monitoring system.

At present, metal oxide semiconductors [7], palladium gate field effect transistors [8], catalytic combustion sensors [9], fuel cell sensors [10,11], and optical sensors [12,13] are the mainly methods applied to detect these characteristic fault gases. Given the remarkable advantages of simple fabrication process, rapid response and recovery time, low maintenance cost and long service life, metal oxide semiconductors such as ZnO [14], SnO_2_[15], TiO_2_[16,17], Fe_2_O_3_[18], NiO [19], WO_3_[20], In_2_O_3_[21] *etc.*, have been widely used for gas sensors. Among these sensing materials, ZnO has attracted increasing attention and been proven to be a highly useful sensing material for detecting both oxidizing and reducing gases [22]. In recent years, great efforts have been made to fabricate low-dimensional ZnO nanostructures [23,24], since their gas sensing properties can be efficiently improved in this way. Taking advantage of their small and uniform particle size, high surface-to-volume ratio, specific pore structure, anti-aggregation properties and so on, these low-dimensional nanostructures may exhibit better sensing properties than those of traditional nanoparticles and thin films.

Hitherto, low-dimensional ZnO nanostructures with different morphologies including nanobelts [25], nanotubes [26], nanorods [27,28], nanowires [29], nanofibers [30], nanodisks [31], nanospindles [31], and nanoneedles [32], have been successfully developed, and many exhibit interesting gas sensing performances towards H_2_, CO, NO_2_, H_2_S, SO_2_ and some volatile organic compounds (VOCs). Pawar *et al.* [31] obtained interesting morphological transformations from rod-to-disk-to-spindle-to-flower merely by varying the pH of the growth solution. Pawar and co-workers [32] also synthesized vertically aligned ZnO nanorods, hexagonal nanorods, faceted microrod rods, nanoneedles and nanotowers assisted with different surfactants (polyetherimide PEI, polyacrylic acid PAA, diammonium phosphate DAP and DAP-PAA). Chai *et al.* [33] reported the synthesis of functionalized individual ZnO microwires prepared by a carbothermal reduction vapor phase transport method and their gas sensing properties for natural gases, such as H_2_, O_2_, CO_2_, CO, CH_4_ and C_2_H_5_OH. Hamedani *et al.* [34] applied a fast and facile microwave assisted method to prepare various ZnO nanocrystal morphologies and investigated their response and selectivity for CO, CH_4_ and C_2_H_5_OH. However, to the best of our knowledge, reports on the hydrothermal synthesis of low-dimensional ZnO nanostructures of various morphologies and their gas sensing properties towards CH_4_ have been rare.

Hence, in this paper we present a simple and effective surfactant-assisted hydrothemal method to synthesize various ZnO nanostructure morphologies, including flower-like ZnO nanorods, net-like ZnO nanofibers and ZnO nanobulks. The additive surfactants are found to play key roles in synthesizing hierarchical flower-like ZnO nanorods and net-like ZnO nanofibers. A possible growth process is discussed in detail and their gas sensing properties towards CH_4_ are measured systematically. The sensors fabricated with ZnO nanorods and nanofibers exhibit better CH_4_-sensing properties than those of nanobulks. These results demonstrate a promising approach to fabricate gas sensors to detect CH_4_ and other characteristic fault gases in power transformers.

## 2. Experimental

### 2.1. Synthsis of Hierarchical ZnO

All the raw chemicals were analytical-grade reagents purchased from Chongqing Chuandong Chemical Reagent Co., Ltd. (Chongqing, China) and used as received without any further purification. Different morphologies of hierarchical ZnO nanostructures, including flower-like ZnO nanorods, net-like ZnO nanofibers and ZnO nanobluks were successfully synthesized via a facile surfactant-assisted hydrothemal method. The detailed synthesis processes were as follows: in a typical synthesis of flower-like ZnO nanorods, Zn(NO_3_)_2_·6H_2_O (0.595 g, 2.0 mmol) and (NH_4_)_2_CO_3_ (0.384 g, 4.0 mmol) were dissolved completely in a mixture of absolute ethanol and distilled water (40.0 mL, 1/1, V/V) in a 100 mL capacity beaker. Then polyvinyl pyrrokidone (PVP, K30, 0.15 g) was added into the beaker. The mixed solution was magnetically stirred for 20 min and transferred into a 100 mL Teflon-lined stainless steel autoclave, sealed and maintained at 180 °C for 24 h. After the hydrothemal reaction was completed , the autoclave was cooled to room temperature naturally. The white products were harvested by centrifugation, washed with distilled water and ethanol four times, respectively, and finally dried at 60 °C in air for 12 h. Finally, the dried products were heated to 400 °C with a rate of 1.0 °C min−1 and annealed at 400 °C for 2 h. The harvest was labeled as S1.

Hierarchical net-like ZnO nanofibers were obtained by the same synthesis process by addition of 0.1 g of polyethylene glycol (PEG, MW = 6,000) to replace the PVP, and it was labeled as S2. For the purpose of comparison, we also prepared samples S3, whose synthesis process is similar to that of the aforementioned process but without adding the PVP or PEG surfactant.

### 2.2. Structure Characterization

The crystalline structures of the prepared products were determined by X-ray powder diffraction (XRD), using a Rigaku D/Max-1200X diffractometer (Tokyo, Japan) with Cu Kα radiation (40 kV, 200 mA and λ = 1.5418 Å), employing a scanning rate 0.02° s−1 with 2θ ranging from 20° to 80°. The morphologies and microstructures were characterized with a Nova 400 Nano field emission scanning electron microscope (FE-SEM, FEI, Hillsboro, OR, USA) operated at 5 kv. The specific surface area and open pore size of the nanostructures were measured by a Surface Area and Porosimetry Analyzer (V-Sorb 2800, Beijing Jinaipu General Instrument Co., Ltd, Beijing, China).

### 2.3. Fabrication and Measurement of Sensors

**Figure 1.**
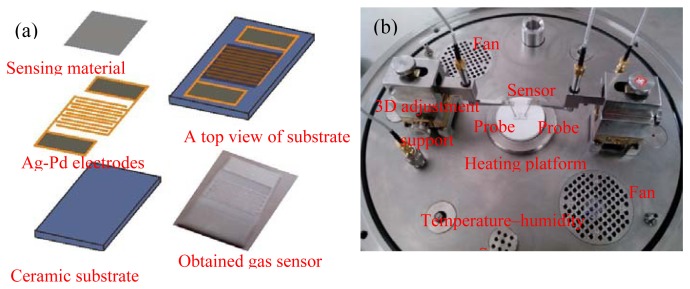
(**a**) Schematic micrograph of the planar ZnO-based gas sensor and obtained gas sensor [inset in (**a**)]; (**b**) A photograph of the gas sensing analysis system.

The ZnO-based gas sensor was fabricated by the screen-printing technique with a planar ceramic substrate, purchased from Beijing Elite Tech Co., Ltd, (Beijing, China). Figure 1(a) shows the schematic and top-view optical micrograph of the planar ZnO-based gas sensor. One can clearly see in Figure 1(a) that the sensor consists of three kinds of significant components: ceramic substrate, Ag-Pd interdigital electrodes, and sensing materials. The bottom layer is the planar ceramic substrate with length, width, and thickness of 13.4, 7, and 1 mm, respectively. Five pairs of Ag-Pd interdigital electrodes were pre-placed on the planar ceramic substrate and the width between electrodes is 0.2 mm. After the as-prepared ZnO samples were further ground into fine powder, it was mixed with diethanolamine and ethanol in a weight ratio of 8:1:1 to form a paste. The paste was subsequently screen-printed onto the planar ceramic substrate to form a sensing film with a thickness of about 50 μm, and then the film was dried in air at 60 °C for 5 h. Finally, the sensor was further aged in an aging test chamber for 48 h.

Gas sensing properties of the as-prepared sensors to CH_4_ were measured by a CGS-1TP (Chemical Gas Sensor-1 Temperature Pressure) intelligent gas sensing analysis system (Beijing Elite Tech Co., Ltd.). The intelligent analysis system was mainly composed by the heating system, circulating cooling system, vacuum system, probe adjustment system, gas distribution system, measurement and data acquisition system, and measurement control software. The heating system and circulating cooling system offered an external temperature control ranging from room temperature to 500 °C with an adjustment precision of 1 °C. As seen in Figure 1(b) the planar sensors were laid on the temperature control and two adjustable probes were pressed on the sensor electrodes to collect electrical signals. When the sensor was pre-heated at certain operating temperature for about 30 min and its resistance was stable, saturated target gas was injected into the test chamber (18 L in volume) by a micro-injector through a rubber plug. The saturated target gas was mixed with air in the test chamber by two fans. After its resistance value reached a new constant value, the test chamber was opened to recover the sensor in air. The sensor resistance and sensitivity were collected and analyzed by the system in real time. The environmental temperature, relative humidity and sensor operating temperature were automatically recorded by the analysis system.

The sensitivity value (S) was designated as S = Ra/Rg, where Ra was the resistance value of prepared planar sensor in air and Rg was that in a mixture of target gas and air. The time taken by the sensor to reach 90% of the total resistance change was defined as the response time in the case of gas adsorption or the recovery time in the case of gas desorption. All measurements were repeated several times to ensure the repeatability and stability of the sensor.

## 3. Results and Discussion

### 3.1. Structural Characterization

**Figure 2.**
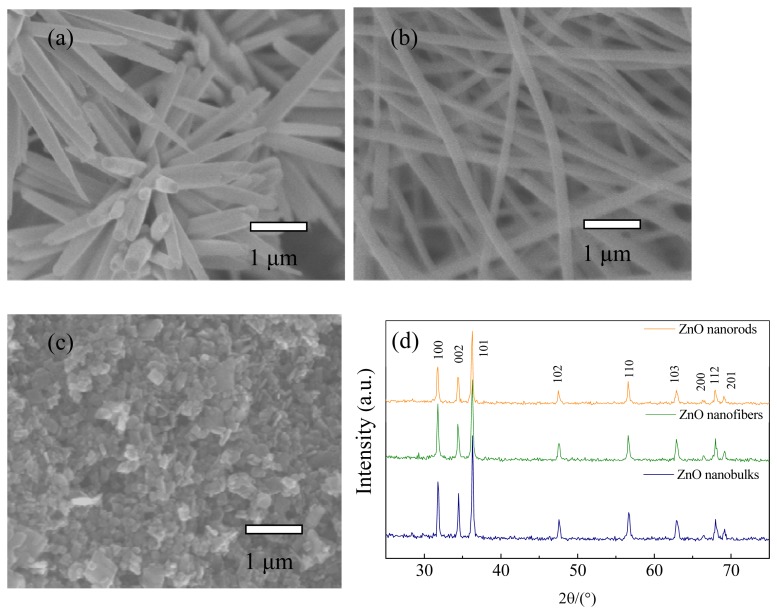
FESEM images of ZnO nanorods (**a**); nanofibers (**b**); nanobulks (**c**) and their XRD patterns (**d**).

To chemically identify the phase composition and purity of the prepared samples, we first conducted the X-ray powder diffraction measurement. Figure 2(d) shows the typical XRD patterns of the as-prepared products. All of the diffraction peaks can be well indexed to the standard diffraction pattern of the wurtzite hexagonal ZnO structure (JCPDS card NO. 36-1451, a = b = 3.249Å and c = 5.206 Å). No diffraction peaks from any other impurities or phases are observed, implying that ZnO crystallites with high purity could be obtained using the current synthetic conditions.

The morphologies and microstructures of the samples synthesized by various methods were further characterized by field emission scanning electron microscopy. Different morphologies of hierarchical ZnO nanostructures, including flower-like nanorods, net-like nanofibers and nanobulks were observed and are shown in Figure 2(a–c). It is clearly seen in Figure 2(a) that the geometrical shapes of ZnO nanorods are rectangular, with high uniformity in shape and size, and the lengths and diameters are estimated to be about 1.5∼2.0 μm and 200∼300 nm, respectively. A large quantity of nanorods self-assembled into flowers. The FESEM images of the hierarchical net-like ZnO nanofibers are shown in Figure 2(b), where one can indeed see the average diameters of each individual nanofiber is about 350 nm with lengths of several microns, and a wide range of ZnO nanofibers joined together and formed a net-like structure. Figure 2(c) displays the FESEM images of S3, where one can see that the products are chaotic and closely packed nanobulks with average lengths, widths and thicknesses of about 400∼500, 200∼300 and 100 nm, respectively. These characterizations demonstrate that we have successfully synthesized the desirable hierarchical ZnO nanostructures via a simple and facile hydrothermal method.

### 3.2. Growth Mechanism

As mentioned above in Figure 2, various morphologies of hierarchical ZnO nanostructures including flower-like nanorods, net-like nanofibers and nanobulks have been successfully synthesized based on different surfactants-assisted hydrothermal synthesis processes. These results indicate that surfactant plays an important role in the final morphologies of the as-prepared products. We think the growth process of various hierarchical ZnO nanostructures can be divided into two stages: the nucleation stage and the self-assembly stage. The former is closely related to the reaction solvents, while the latter is mainly depends on the surface-active agents. A certain amount of Zn(OH)_4_^2−^ ions dissolved in aqueous solution has been reported in paper [35] as a prerequisite for hydrothermal synthesis of hexagonal ZnO nanostructures. The chemical reactions for the formation of the ZnO precursor in our programs can be expressed as follows:

$$\begin{array}{cc} {\left({\text{NH}}_{4}\right)}_{2}{\text{CO}}_{3}\to {2{\text{NH}}_{4}}^{-}+{{\text{CO}}_{3}}^{2-} & {{\text{NH}}_{4}}^{-}+{\text{H}}_{2}\text{O}\to {\text{NH}}_{3}+{\text{H}}_{2}+{\text{OH}}^{-}\\ {{\text{CO}}_{3}}^{2-}+{\text{H}}_{2}\text{O}\to {{\text{HCO}}_{3}}^{-}+{\text{OH}}^{-} & {{\text{HCO}}_{3}}^{-}+{\text{H}}_{2}\text{O}\to {\text{H}}_{2}{\text{CO}}_{3}+{\text{OH}}^{-}\\ {\text{Zn}}^{2+}+4{\text{OH}}^{-}\to {{\text{Zn(OH)}}_{4}}^{2-} & {{\text{Zn(OH)}}_{4}}^{2-}\to {\text{ZnO}+\text{H}}_{2}{\text{O}+2\text{OH}}^{-} \end{array}$$

**Figure 3.**
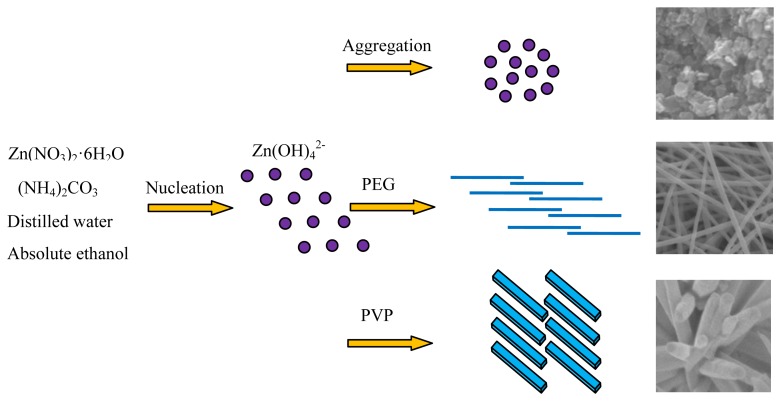
Schematic diagrams of the formation process of ZnO nanorods, nanofibers and nanobulks.

The self-assembly mechanism of ZnO nanostructures with different morphologies were diverse when various surfactants were used. In order to further understand how the additive surfactants affect the morphologies of prepared ZnO architectures, a series of possible self-assembly processes were described in detail by the following schematic diagram shown in Figure 3.

As is known to all that PVP is a non-ionic surfactant, and an easily polarized functional group “–C=O” is universally present in its repeated unit [36,37]. The “O” atom in the “–C=O” functional group of PVP has a negative charge and the “Zn” in the precursor particles are positively charged, so an intense attraction occurs between “O” and “Zn” atoms when PVP was added to the mixed solution in our experiments, and ZnO nanorods were produced. Correspondingly, these ZnO nanorods aggregated and self-assembled toward the common centre of PVP. With the increase of the aggregation, finally hierarchical flower-like ZnO nanostructures appeared.

PEG is a kind of template with long chains, and numerous of hydrophilic “–O–“ and “–CH_2_–CH_2_–“ groups exist along its long chains. When PEG was added to the mixed solution, it worked as a assembly substrate, which can easily embed Zn(OH)_4_^2−^ in its long-chain orientation. Under the current hydrothermal program, numerous tiny Zn(OH)_4_^2−^ crystals nucleated on the PEG long chains and grew [38,39]. As the reaction time went by, the Zn(OH)_4_^2−^ crystals gradually grew with a fiber-like structure along the long chains of PEG and novel three-dimensional net-like ZnO nanostructures were finally synthesized. When no surfactant was added to the solvent, the Zn(OH)_4_^2−^ precursors nucleate and aggregate chaotically, and bulk-like ZnO nanostructures were formed.

### 3.3. Methane Sensing Properties

**Figure 4.**
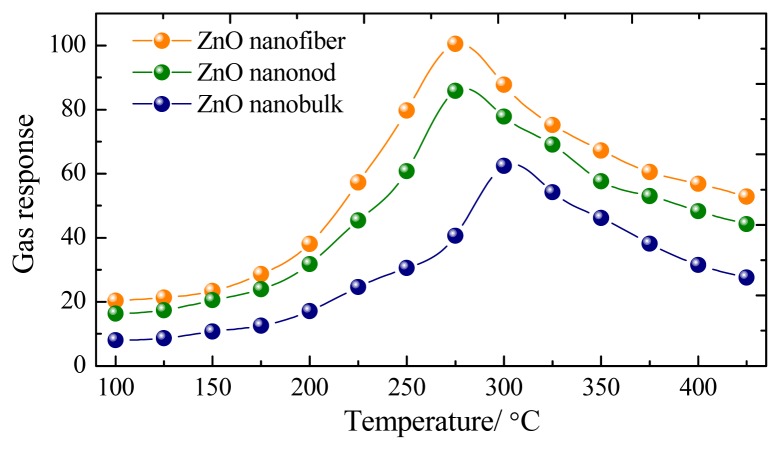
Gas response of the sensors to 200 μL/L of CH_4_ at different operating temperature.

Three kinds of gas sensors were fabricated with the prepared ZnO nanorods, nanofibers and nanobulks. Gas sensing experiments of these sensors towards CH_4_ were first performed at different operating temperatures to find out the optimum working temperature. Figure 4 indicates the gas response of the sensor to 200 μL/L of CH_4_ as a function of operating temperature ranging from 100 to 425 °C. As shown in Figure 4, the optimum operating temperature of the prepared nanorods and nanofibers sensors to CH_4_ gas are both about 275 °C, and it is 300 °C for nanobulks sensor, where the sensor exhibits the maximum gas response.

**Figure 5.**
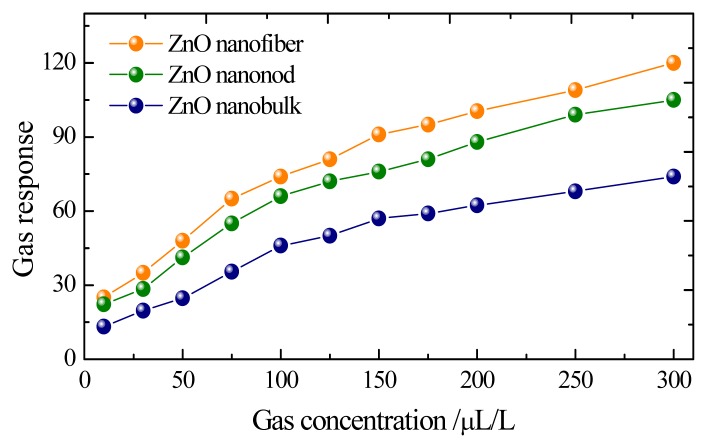
Gas response of the sensors to different concentration ranking from 10 to 300 μL/L.

Gas responses of these sensors to different concentrations of CH_4_ are shown in Figure 5, with the sensors working at their optimum operating temperature as mentioned above. As seen in Figure 5 the sensors based on ZnO nanorods and nanofibers exhibit much higher sensitivities than that of nanobulk sensors to the same concentration of CH_4_ gas in all measurements. The gas responses to 10, 50, 100 and 200 μL/L of CH_4_ are only about 13.25, 24.71, 46.18 and 62.38 for the bulk-shaped ZnO sensor, while they are about 22.25, 41.94, 66.08 and 88.27 for the rod-shaped one and 25.33, 48.27, 74.35 and 100.49 for the fiber-shaped ZnO sensor, respectively. Meanwhile, the quasi-linear response curves imply these prepared sensor can be applied for engineering applications.

**Figure 6.**
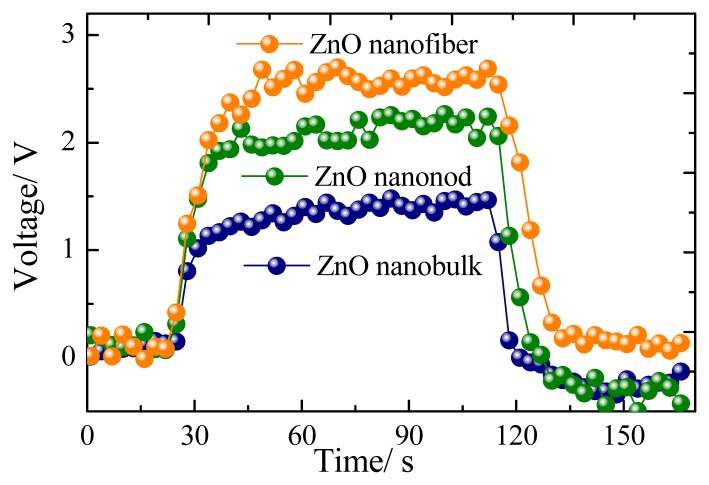
The response-recovery curves of the sensors to 100 μL/L of CH_4_.

**Figure 7.**
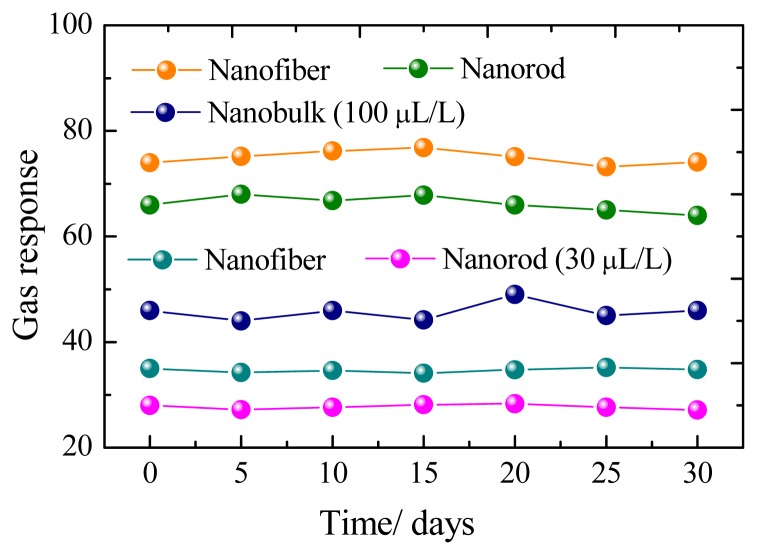
Stability and repeatability of the sensors to 100 and 30 μL/L of CH_4_.

Figure 6 presents a representative response-recovery curve of the sensor to 100 μL/L of CH_4_ with the sensor operating at its optimum operating temperature. According to the description above, the response and recovery times for the nanorods, nanofibers and nanobulks are evaluated to be about 13–6 s, 14–8 s and 18–9 s, respectively. The long-time stability of the three kinds of sensors has been also measured, as shown in Figure 7. It can be clearly seen that the rod-shaped and fiber-shaped ZnO sensors exhibit nearly constant voltage values during the long experimental cycles, while higher fluctuations are observed for bulk-shaped ZnO sensors. These results confirm the long-term stability and good repeatability of the prepared flower-like ZnO nanorods and net-like ZnO nanofibers gas sensors.

In the light of the measurements above, we can conclude that low-dimensional ZnO nanostructures including nanorods and nanofibers exhibit better sensing properties than that of the nanobulks. Further understanding of these sensing properties can be performed by analyzing the gas sensing reaction process and the structural characteristics of the nanocrystals.

It is well known that ZnO is a typical n-type semiconductor gas sensing material, and its gas sensing properties are dominated by the surface resistance. In ambient air, oxygen would be absorbed on the ZnO surface at first. Due to its strong electronegativity, absorbed oxygen then acts as a trap capturing electrons from the conduction band of ZnO. Consequently, a depletion region on the surface appears, resulting in an increase in the ZnO resistance. When the sensor is exposed to ambient CH_4_, the CH_4_ molecules react with adsorbed oxygen and trapped electrons are released back to the conduction band, thus an decreased resistance is measured. The entire adsorption and reaction process can be expressed as follows [40]:

$$\begin{array}{l} {\text{O}}_{2}\left(\text{gas}\right)\to {\text{O}}_{2}\left(\text{ads}\right)\;{\text{O}}_{2}\left(\text{ads}\right)+{\text{e}}^{-}\to {{\text{O}}_{2}}^{-}\left(\text{ads}\right)\\ {{\text{O}}_{2}}^{-}\left(\text{ads}\right)+{\text{e}}^{-}\to 2{\text{O}}^{-}\left(\text{ads}\right)\;{\text{O}}^{-}\left(\text{ads}\right)+{\text{e}}^{-}\to {\text{O}}^{2-}\left(\text{ads}\right)\\ {\text{CH}}_{4}\left(\text{gas}\right)+{2{\text{O}}_{2}}^{-}\left(\text{ads}\right)\to {\text{CO}}_{2}\left(\text{gas}\right)+2{\text{H}}_{2}\text{O}\left(\text{gas}\right)+2{\text{e}}^{-}\\ {\text{CH}}_{4}\left(\text{gas}\right)+4{\text{O}}^{-}\left(\text{ads}\right)\to {\text{CO}}_{2}\left(\text{gas}\right)+2{\text{H}}_{2}\text{O}\left(\text{gas}\right)+4{\text{e}}^{-}\\ {\text{CH}}_{4}\left(\text{gas}\right)+4{\text{O}}^{2-}\left(\text{ads}\right)\to {\text{CO}}_{2}\left(\text{gas}\right)+2{\text{H}}_{2}\text{O}\left(\text{gas}\right)+8{\text{e}}^{-} \end{array}$$

Compared with tranditional ZnO nanobulks, ZnO nanorods and nanofibers exhibit much higher gas responses at a low temperature with rapid response-recovery times, long-term stability and good repeatablity. These can be explained with the hierarchical ZnO nanostructures and morphologies formed by flower-like nanorods and net-like nanofibers. The main factors that influence the gas sensing behaviors of the materials are the specific surface area and pore structure.

**Figure 8.**
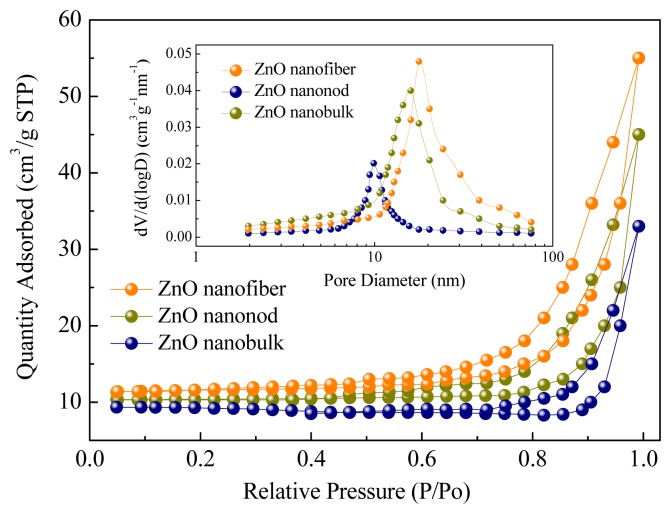
Nitrogen adsorption-desorption isotherms of ZnO nanorods, nanofibers and nanobulks. The inset to the figure shows the corresponding pore size distribution curves.

To further confirm the inner architectures of the as-prepared ZnO nanostructures, nitrogen adsorption and desorption measurements were performed to estimate the texture properties. The representative nitrogen adsorption-desorption isotherm and the corresponding Barrett–Joyner–Halenda (BJH) [41,42] pore size distribution plot (inset) of the nanostructures are shown in Figure 8. Using the BJH method and the desorption branch of the nitrogen isotherm, the calculated BET surface area and pore structure parameters are shown in Table 1. As shown, the measured surface area for the nanofibers, nanorods and nanobulks are 28.6, 26.4 and 19.7 m^2^/g, and the surface pore sizes are 18, 16 and 10 nm, respectively. Such hierarchical morphologies, together with the larger scale of the surface area and pores, will form much more depletion regions around the intersection and surface. Thus more efficient charge transfer take place on the surface and excellent gas sensing properties are observed in our experiments.

## 4. Conclusions

A simple hydrothemal method was developed to synthesize ZnO nanostructures of different morphologies, including flower-like nanorods, net-like nanofibers and nanobulks. X-ray powder diffraction and field emission scanning electron microscopy were used to determine the phase compositions and morphologies of the samples. A possible growth mechanism of the synthesized nanostructures was discussed in detail and their gas sensing properties towards CH_4_ were systematically measured. The sensors based on hierarchical ZnO nanorods and nanofibers exhibit better gas sensing performance features, such as higher gas response, lower operating temperature, rapid response-recovery time, long-term stability and repeatablity than ZnO nanobulks. Such results indicate that the as-synthesized ZnO nanorod and nanofiber-based sensors are promising candidates for on-site detection of characteristic fault gases dissolved in transformer oil.

## Figures and Tables

**Table 1.** BET surface area and pore structure parameters of the samples.

**ZnO Nanostructures**	**S_BET_ (m^2^g^−1^)**	**d_P_ (nm)**
Nanofibers	28.6	18
Nanorods	26.4	16
Nanobulks	19.7	10
